# Supplementary figures and images for: T-dependent B cell responses to Plasmodium induce antibodies that form a high-avidity multivalent complex with the circumsporozoite protein
Source: PLoS Pathog. 2017 Jul 31;13(7):e1006469. doi: 10.1371/journal.ppat.1006469 (PMC5552345; doi:10.1371/journal.ppat.1006469)

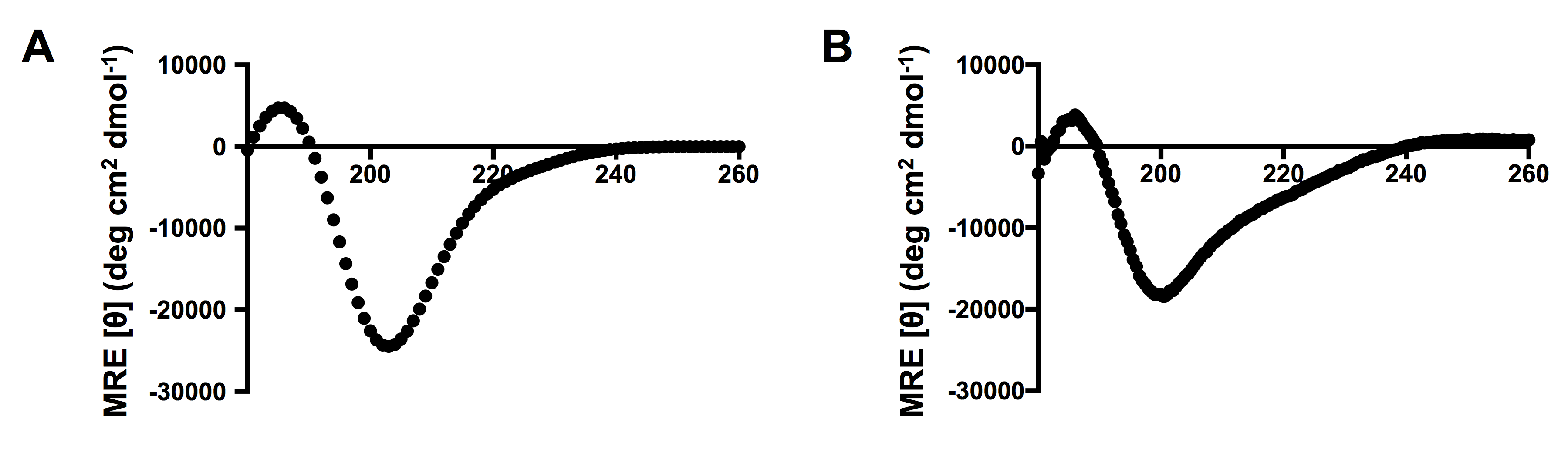

Supplement: S1 Fig — The computational prediction of the spectra (A) was performed using DichroCalc [57], the experimental spectra was measured at 222 nm at 25°C. A peak at 185 nm, minimum at 205 nm and shoulder between 215 and 240 nm are consistent with an intrinsically disordered, but not random coil, structure. (TIF) [file ppat.1006469.s001.tif]

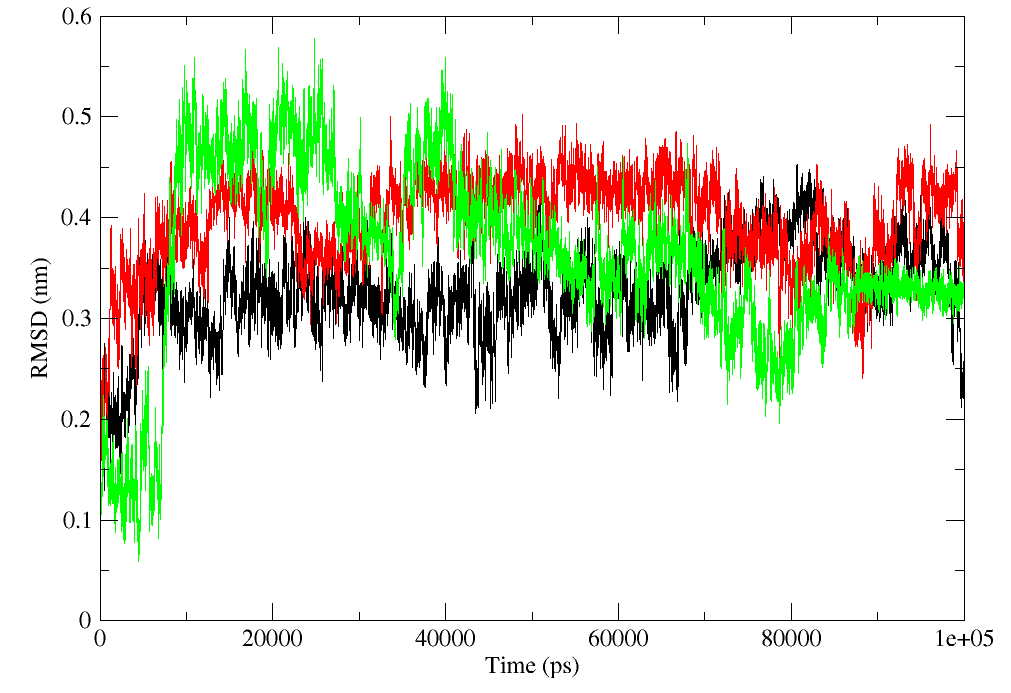

Supplement: S3 Fig — Molecular dynamics simulation of the (NANP)6:FAB complex. Root mean square deviation (RMSD) of the (NANP)6:FAB complex as a function of time. Independent simulations are shown in green, black and red. (TIF) [file ppat.1006469.s003.tif]

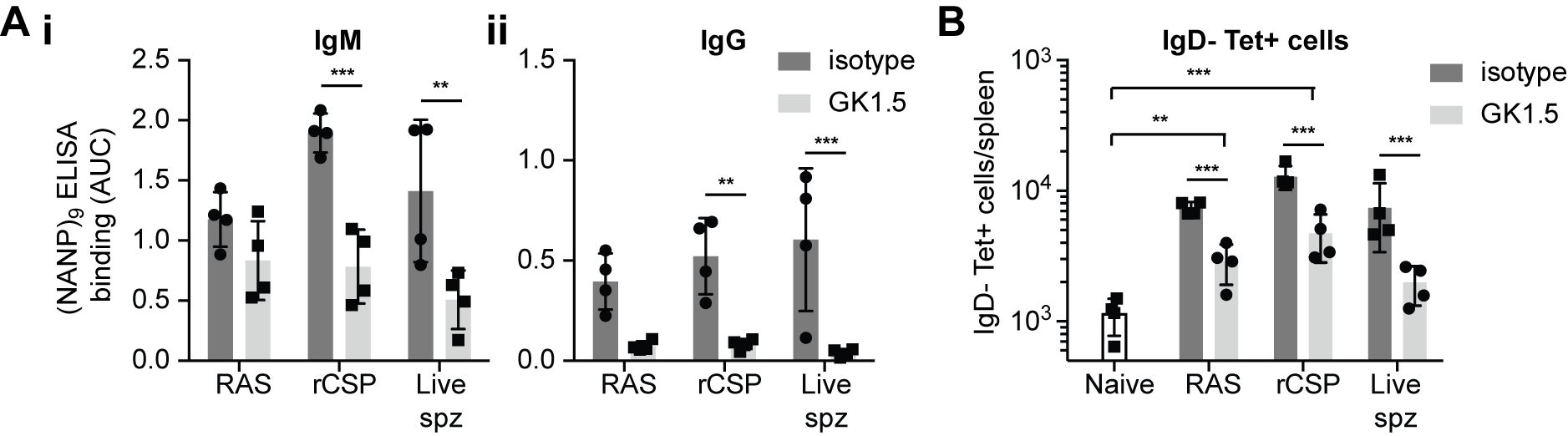

Supplement: S4 Fig — Mice either treated with an anti-CD4 depleting antibody or an isotype control were immunizaed with either P. berghei CSPf RAS, live P. berghei CSPf under CQ cover or rCSP. (A) 4 days later the IgM and IgG response to the (NANP)n repeat was analyzed by ELISA (B) At the same time the number of IgD- Tetramer+ B cells was quantified in the spleen. Data are from a single experiment, analyzed using linear models with immunization/treatment as the experimental factor. (TIF) [file ppat.1006469.s004.tif]

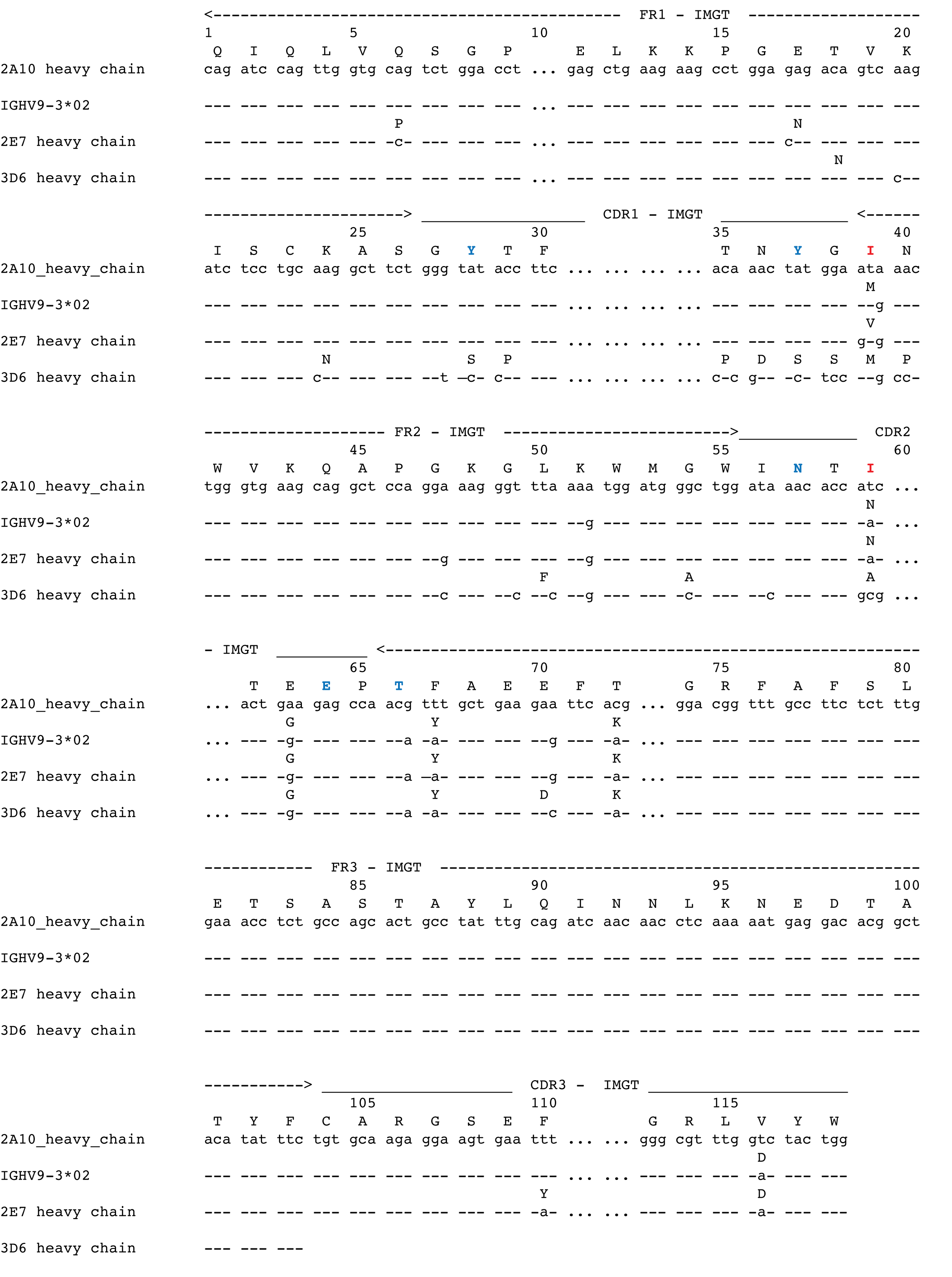

Supplement: S5 Fig — Residues that are mutated away from the predicted germline sequence in more one or more other antibody heavy chain (2E7 or 3D6) are highlighted in red, mutations that are predicted to be involved in binding to CSP are highlighted in blue. (TIF) [file ppat.1006469.s005.tif]

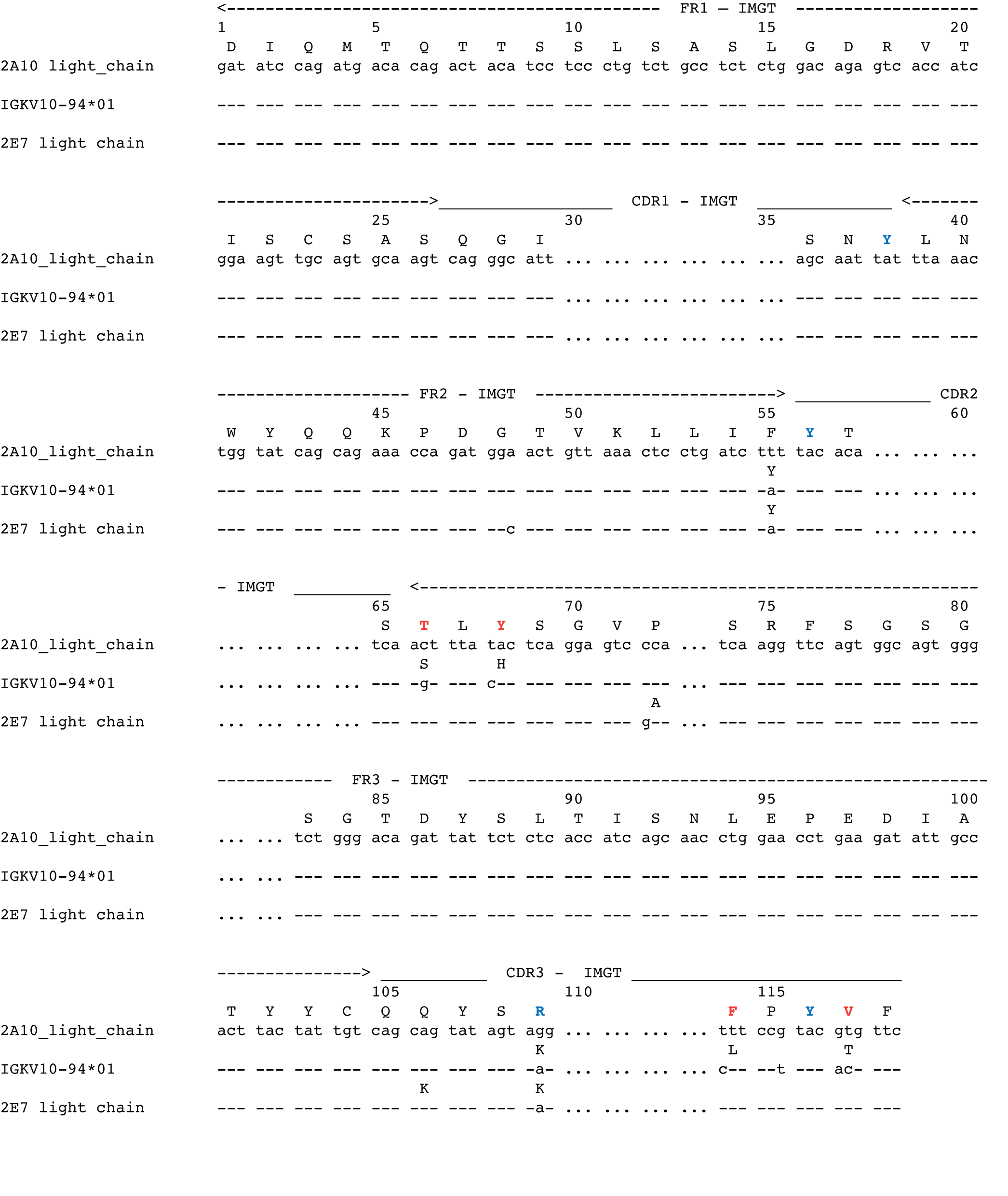

Supplement: S6 Fig — Residues that are mutated away from the predicted germline sequence in both 2A10 and the related 2E7 antibody are highlighted in red, mutations that are predicted to be involved in binding to CSP are highlighted in blue. (TIF) [file ppat.1006469.s006.tif]
