# Supplementary material for: T-dependent B cell responses to Plasmodium induce antibodies that form a high-avidity multivalent complex with the circumsporozoite protein
Source: PLoS Pathog. 2017 Jul 31;13(7):e1006469. doi: 10.1371/journal.ppat.1006469 (PMC5552345; doi:10.1371/journal.ppat.1006469)
Supplement: S1 Table — (DOCX) [file ppat.1006469.s009.docx]

**S1 Table. Data collection and refinement statistics for the crystal structures of 2A10 F_AB_ presented in this work.**

|  | 2A10 F_AB_ 2.52 | 2A10 F_AB_ 3.01 |
| --- | --- | --- |
| **PDB ID** | 5SZF | 5T0Y |
| **Data collection** |  |  |
| Space group | I4_1_32 | P432_1_2 |
| Cell dimensions |  |  |
| *a*, *b*, *c* (Å) | 204.21 | 231.68, 231.68, 81.78 |
|    γ (°) | 90 | 90 |
| Resolution (Å) | 37.28-2.52  (2.62-2.52) | 39.73-3.01  (3.12-3.01) |
| *R*_merge_ | 0.178 (1.836) | 0.315 (1.913) |
| *R*_pim_ | 0.028 (0.406) | 0.085 (0.517) |
| CC_1/2_ | 0.999 (0.620) | 0.991 (0.718) |
| Completeness (%) | 100 (100) | 99.9 (99.5) |
| Redundancy | 39.2 (22.0) | 14.6 (14.6) |
|  |  |  |
| **Refinement** |  |  |
| Resolution (Å) | 37.28-2.52  (2.61-2.52) | 39.73-3.01  (3.12-3.01) |
| No. reflections | 24796 (2440) | 44596 (4352) |
| *R*_work_ / *R*_free_ | 0.2251/0.2483 | 0.2248/0.2467 |
| No. atoms |  |  |
| Protein | 3288 | 9856 |
| Ligand/ion | 20 | 90 |
| Water | 46 | 25 |
| Wilson *B*-factor | 46.80 | 60.66 |
| R.m.s. deviations |  |  |
| Bond lengths (Å) | 0.003 | 0.002 |
| Bond angles (°) | 0.57 | 0.55 |
| Ramachandran favored (%) | 95 | 95 |
| Ramachandran outliers (%) | 0 | 0 |

*Values in parentheses are for highest-resolution shell.
