# Supplementary material for: T-dependent B cell responses to Plasmodium induce antibodies that form a high-avidity multivalent complex with the circumsporozoite protein
Source: PLoS Pathog. 2017 Jul 31;13(7):e1006469. doi: 10.1371/journal.ppat.1006469 (PMC5552345; doi:10.1371/journal.ppat.1006469)
Supplement: S2 Table — (DOCX) [file ppat.1006469.s010.docx]

**S2 Table: Heavy chain CDR sequences of CSP binding antibodies**

| **Antibody** | **Species** | **Heavy chain** | **CDR1** | **CDR1 group** | **CDR2** | **CDR2 group** | **CDR3** | **CDR3 group** |
| --- | --- | --- | --- | --- | --- | --- | --- | --- |
| 2A10 | Mouse | HV9-3*02 | KASG**Y**TF....TN**Y**G**I**N* | H1-13-1 | WI**N**T**I**..TEEP**T** | H2-10-1 | ARGSEFGRLVY | N/A |
| PfNPNAI | Human | HV3-30-3 | AASGFTF....SSYAMH | H1-13-1 | VISYD..GSNKY | H2-10-2 | DRDSSSYFDS | N/A |
| 3D6 | Mouse | HV9-2-1*01 | KASGSPF....PDSSMP | H1-13-1 | WINTA..TGEPT | H2-10-1 | GGGGGPWFAY | N/A |
| 2C11 | Mouse | HV1-20 OR 37 | KASGYSF....TGSFMN | H1-13-1 | RINPN..DGYTF | H2-10-1 | GKGNHGATDY | N/A |
| 1E9 | Mouse | HV1-20 OR 37 | KASGYSF....TGSFMN | H1-13-1 | RILPY..NGDTF | H2-10-1 | GYVYDGGYATDY | N/A |
|  |  |  |  |  |  |  |  |  |
|  | Mouse | HV5-9 | AASGFTF....SSYTMS | H1-13-1 | TISSG..GGNTY | H2-10-1 | VARIABLE |  |
|  | Mouse | HV1-20 | KASGYSF....TGYFMN | H1-13-1 | RINPY..NGDTF | H2-10-1 | VARIABLE |  |
|  | Mouse | HV1-26 | KASGYTF....TDYYMN | H1-13-1 | DINPN..NGGTS | H2-10-1 | VARIABLE |  |
|  | Mouse | HV1-34 | KASGYTF....TDYYMH | H1-13-1 | YIYPN..NGGNG | H2-10-1 | VARIABLE |  |

*Letters in bold denote resides show to be required for binding
