# Supplementary material for: T-dependent B cell responses to Plasmodium induce antibodies that form a high-avidity multivalent complex with the circumsporozoite protein
Source: PLoS Pathog. 2017 Jul 31;13(7):e1006469. doi: 10.1371/journal.ppat.1006469 (PMC5552345; doi:10.1371/journal.ppat.1006469)
Supplement: S3 Table — (DOCX) [file ppat.1006469.s011.docx]

| **Antibody** | **Species** | **Light chain** | **CDR1** | **CDR1 group** | **CDR2** | **CDR2 group** | **CDR3** | **CDR3 group** |
| --- | --- | --- | --- | --- | --- | --- | --- | --- |
| 2A10 | Mouse | KV10-94 | SASQGI......SN**Y**LN* | L1-11-2 | F**Y**TSTL**Y**S | L2-8-1 | QQYS**R**F**PYV** | L3-9-cis7-1 |
| PfNPNAI | Human | KV1-5 | RASQSI......SSWLA | L1-11-2 | YDASSLES | L2-8-1 | QQYNSYSGLT | L3-10-1 |
| 3D6 | Mouse | KV6-20 | KASENV......VTYVS | L1-11-1 | YRASNRYT | L2-8-1 | GQGSSYPYT | L3-9-cis7-1 |
| 2C11 | Mouse | KV5-43 | RASQNI......SNNLH | L1-11-1 | TYASQSIS | L2-8-1 | QQSNSWPLT | L3-9-cis7-1 |
| 1E9 | Mouse | KV1-110 | RSSQSLGHS.HGNTYLH | L1-16-1 | YKVSNRFS | L2-8-1 | SQSTQLRT | L3-8-1 |
|  |  |  |  |  |  |  |  |  |
| High | Mouse | KV1-117 | RSSQSIVHS.NGNTYLE | L1-16-1 | YKVSNRFS | L2-8-1 | FQGSHVPPTF | L3-9-cis7-1 |
| Throughput | Mouse | KV1-110 | RSSQSLVHS.NGNTYLH | L1-16-1 | YKVSNRFS | L2-8-1 | SQSTHLPLTF | L3-9-cis7-1 |
| Sequencing | Mouse | KV1-135 | KSSQSLLDS.DGKTYLN | L1-16-1 | YLVSKLDS | L2-8-1 | WQGTHFPFTF | L3-9-cis7-1 |
|  | Mouse | KV5-43 | RASQSI......SNNLH | L1-11-1 | KYASQSIS | L2-8-1 | QQSNSWPLTF | L3-9-cis7-1 |
|  | Mouse | KV5-45 | RASQSI......SNYLH | L1-11-1 | KYASQSIS | L2-8-1 | QQSNSWPLTF | L3-9-cis7-1 |
|  | Mouse | KV14-111 | KASQDI......NSYLS | L1-11-1 | YRANRLVD | L2-8-1 | LQYGEFPPTF | L3-9-cis7-1 |

**S3 Table: Light chain CDR sequences of CSP binding immunoglobulins**

*Letters in bold denote resides show to be required for binding
