# Supplementary material for: T-dependent B cell responses to Plasmodium induce antibodies that form a high-avidity multivalent complex with the circumsporozoite protein
Source: PLoS Pathog. 2017 Jul 31;13(7):e1006469. doi: 10.1371/journal.ppat.1006469 (PMC5552345; doi:10.1371/journal.ppat.1006469)
Supplement: S1 Methods — (DOCX) [file ppat.1006469.s012.docx]

**S1 Methods: Supplementary Methods**

## Generation of antibody variants

Constructs containing minigenes for the monoclonal and germline heavy (isotype: IgG2A) and light chains of the 2A10 antibody in a pcDNA3.1+ backbone were ordered commercially (Biomatik). Mutations described in the figure legends were introduced using the QuikChange II site directed mutagenesis kit according to the manufacturers instructions (Agilent). To generate antibodies HEK293T cells grown in DMEM supplemented with Nutridoma-SP (Roche) were transfected with 15 μg of each of the heavy and light chain plasmids in 0.06mg/ml branched PEI in 120mM NaCl. 3 and 6 days following transfections supernatants were collected, concentrated over a 100kDa Ultra-15 centrifugal filter unit, Ultracell-100 membrane (Amicon). Antibody concentrations were determined by sandwich ELISA on coats plated with anti-mouse kappa (Southern Biotech) as capture antibodies and horseradish peroxidase conjugated anti-mouse IgG2A (KPL) as detection antibodies.

## ELISA

Binding of 2A10 antibody variants was determined in solid phase ELISA. Briefly, Nunc Maxisorp Plates (Nunc-Nucleon) were coated overnight with 1ug/ml streptavidin followed by binding of biotinylated (NANP)_9_ peptide for 1 hour. After blocking with 1% BSA, serial dilutions of the antibodies were incubated on the plates for 1 hour and after washing, incubated with HRP conjugated anti-IgG2A antibodies (KPL). For the analysis of sera from immunized mice data were expressed as the area under the curve (AUC) which was calculated in Genstat, using the log(dilution) on the x axis and the Absorbance at 405nm on the y axis. The mean AUC from a group of naïve control mice in each experiment was subtracted from the AUC of each immunized mouse to remove background.

## High throughput sequencing of (NANP)_n_ specific B cell receptors

RNA was extracted using the Arcturus Picopure RNA isolation kit and cDNA prepared using the iScript cDNA synthesis kit (Biorad) according to the manufacturer’s instructions. BCR sequences were amplified using previously described heavy and kappa chain primers including adaptor sequences allowing subsequent indexing using the Nextera indexing kit (Illumina). Amplification conditions were 1 cycle at 95°C for 5 minutes followed by 50 cycles at 95°C for 1 minute, 43°C for 1 minute and 72 for 1.5 minutes then finally 1 cycle at 72°C for 5 minutes before holding at 4°C to cool. Following initial amplification PCR products were cleaned up using AMpure XP beads (Beckman Coulter) according to the manufacturer’s instructions. Subsequently the cleaned up libraries were used as templates for the indexing step using the Nextera indexing kit (Illumina). Indexing PCR was performed using the following setting 72 °C for 30 seconds then 95 °C for 30 seconds followed by 15 cycles of 95 °C for 15 seconds, 63 °C for 30 seconds then finally 73 °C for 3 minutes before holding at 4 °C. Samples are then cleaned up for a second time using the AMpure XP beads. The amount of each library was determined using a Caliper™ GX II and 5 μL of each library at 2 nM was pooled sequenced using the Illumina MiSeq sequencer performing 2x 300bp paired reads.

## Sequencing analysis

Trimmomatic was used to clean up and remove unwanted paired end forward and reverse reads from the raw Fastq files generated by the MiSEQ. This involved cutting the Nextera sequencing adaptors from the read, cutting bases from the start or the end of the read if they had a quality score lower then 3, removing reads when the average quality within a window of 4 base pairs drops below a quality score of 20 and removing any reads below 150 bp (for kappa chain reads) or 50bp for heavy chain reads were dropped. The program MiXCR [[1](#_ENREF_1)] was then used to analyze the cleaned paired end forward and reverse files. Forward and reverse reads were t aligned to known mouse V(D)J genes using the default align command. From these alignments clonotypes were built using MiXCR’s assemble command based on the CDR3. For kappa chains, additional clonotypes was built based of the entire VJ transcript. These clonotypes were exported into .txt files using the export command. Further analysis into VDJ usage and diversity was done using the R package tcR [[2](#_ENREF_2)]. SHM analysis was done using in-house scripts to analyze the data generated by the best V (and J) sequences.

**Protein purification**

2A10 and 2A10 F_AB_ fragment were produced from hybridomas by Genscript (Piscataway, NJ) and purified using Protein A before being resuspended in PBS with 0.02% Sodium Azide and shipped as a lyophilized powder. His tagged rCSP was expressed in *E.coli* by Genscript (Piscataway, NJ) and purified from the supernatant of the cell lysate prior to being shipped in PBS with 10% Glycerol. Prior to ITC, CD and X-ray crystallographic analysis, 2A10, 2A10 F_AB_ fragment and rCSP were purified by size-exclusion chromatography using a HiLoad 26/600 Superdex 200 column (GE Healthcare). 2A10 and the 2A10 F_AB_ fragment were eluted in 25 mM TRIS pH 7.2, 100 mM NaCl. rCSP was eluted in 50 mM TRIS pH 7.2, 200 mM NaCl, and then transferred into 25 mM TRIS pH 7.2, 100 mM NaCl using a PD 10 desalting column (GE Healthcare) immediately prior to ITC experiments. Protein purity was confirmed using SDS-PAGE.

## Isothermal Titration Calorimetry.

ITC experiments were performed using a Nano-ITC low volume calorimeter (TA Instruments) at 25 °C, with stirring at 250 rpm. Protein solutions were prepared in TRIS buffer and degassed before use. For the F_AB_-(NANP)_6_ titration, 50 µM 2A10 F_AB_ was titrated with 1 × 1.2 µL, then 20 × 2.0 µL injections of 400 µM (NANP)_6_. For the F_AB_-CSP titration, 8.1 µM 2A10 F_AB_ was titrated with 1 × 1.2 µL, then 20 × 2.0 µL injections of 5.9 µM CSP. For the 2A10-CSP titration, 8.8 µM 2A10 was titrated with 1 × 1.2 µL, then 28 × 1.5 µL injections of 9.0 µM CSP. Data were analyzed in NanoAnalyze software (TA Instruments); the baseline-subtracted power was integrated, and the integrated heats were fit to the independent binding sites model to obtain the stoichiometry of the interaction (*n*), the association constant (*K*_a_), and the enthalpy of binding (ΔH). The background heat was included as an adjustable parameter in the model. 95% confidence intervals for *n*, *K*_a_ and ΔH were estimated by simulating 500 replicate datasets and fitting them to the independent binding sites model, as implemented in NanoAnalyze software.

**Protein crystallography**

The 2A10 F_AB_ fragment was concentrated to either 15 or 24 mg/mL using 100 kDa centrifugal filter units (Millipore). High throughput crystallisation screens were set up at the C3 crystallization facility, CSIRO (Melbourne). Crystals formed in conditions of 2 M ammonium sulfate, 0.1 M trisodium citrate (condition A), pH 5.5 and 2 M ammonium sulfate, 0.1 M bis-tris chloride, pH 6.5 (condition B). Crystals were added to cryo buffer (reservoir conditions with addition of 35% glycerol) and flash-cooled in liquid nitrogen. X-ray diffraction data were collected the MX1 beamline of The Australian Synchrotron. Crystals from condition A crystallized in the I4_1_3 2 space group and diffracted to 2.52 Å with one F_AB_ monomer in the asymmetric unit. Crystals from condition B crystallized in the P4_3_2_1_2 spacegroup and diffracted to 3.01 Å with three F_AB_ monomers in the asymmetric unit. The structures were solved by molecular replacement using PHASER with the PDB ID: 2BRR as the search model for the heavy chain and PDB ID: 1EMT [[3-5](#_ENREF_3)] as the search model for the light chain. Iterative cycles of manual model building and refinement were performed using Coot 0.8.2 [[6](#_ENREF_6)]and phenix.refine [[7](#_ENREF_7)]. Coordinates and structure factors were deposited in the Protein Data Bank with accession codes 5ZSF (condition A) and 5T0Y (condition B).

**Circular dichorism**

To determine the solution structure of the (NANP)_6_ peptide, far-UV CD was utilized. The peptide was diluted in 100 mM NaCl, 25 mM Tris, pH 7.2 to a concentration of 0.2 mg/mL and scanned from 180 - 260 nm in 0.5 nm steps at 20 °C on an Applied Photophysics ChiraScan circular dichroism spectrometer. The structure of the peptide was predicted using the PEP-FOLD *de novo* peptide structure prediction server using default settings [[8](#_ENREF_8)]. Only one low energy structure exhibited repeating order; this structure was then used to calculate the predicted CD spectrum using DichroCalc considering 2 backbone charge transitions, side chain transitions, and with an ab initio parameter set [[9](#_ENREF_9)].

**Computational modelling of the 2A10:(NANP)_6_ interaction**

The 2.52 Å structure of the 2A10 F_AB_ fragment and the ab initio predicted structure of the (NANP)_6_ peptide were used to model of the complex. First, an initial approximate model was generated using the GRAMM-X protein:protein docking web server [[10](#_ENREF_10)], using default settings. The best model from the GRAMM-X output was then used as input for Rosetta SnugDock [[11](#_ENREF_11)], again using default parameters. To model the full complex, the (NANP)_6_ peptide structure was duplicated and partially superimposed, to extend it to 27 repeats. The complex between the 2A10 F_AB_ fragment and an epitope was then overlaid in a repeating fashion along the repeating unit. Superposition was carried out using Pymol 1.8.2.3 (Schrodinger, LLC, USA).

For molecular dynamics simulations, both the (NANP)_6_ peptide structure and each peptide in the 2A10:(NANP)_6_ complex models were capped with acetyl and amine groups. The peptide was solvated in SPC water in a truncated dodecahedral box with a distance of 5 nm between periodic images to allow the peptide some flexibility before encountering its periodic image. Meanwhile, the 2A10:(NANP)_6_ complex was solvated in a truncated dodecahedral box with a distance of 3 nm between periodic images. Sodium and chloride ions were added to both systems to make up 200 mM salt solutions. All simulations were performed with GROMACS 5.1.2 [[12](#_ENREF_12)]in the GROMOS 54A7 forcefield [[13](#_ENREF_13)] on an in-house compute server with 2 Nvidia Tesla K20 GPUs and 32 CPU cores. Long-range electrostatics were treated with the Particle Mesh Ewald method and the Van der Waals cut-off was set to 1.4 nm. The temperature was coupled to a virtual water bath at 300 K with a velocity rescale thermostat. The Berendsen barostat was used during equilibrations with a time constant of 2 fs; production runs were pressure coupled with a Parrinello-Rahman barostat with a time constant of 10 fs. A 2 fs time step was used throughout. Simulations were initially equilibrated with a 1 ns (500 000 steps) simulation in which alpha carbons were position restrained with a force constant of 1000 kJ mol^-1^ nm^-1^. The position restraints were relaxed over a series of 5 further 1 ns equilibrations with restraints of 500, 100, 50, 10 and 0 kJ mol^-1^ nm^-1^. Finally, production runs were performed for 100 ns (50 000 000 steps). Equilibration and production simulations were performed in triplicate for each system.

**Supplementary References**

1. Bolotin DA, Poslavsky S, Mitrophanov I, Shugay M, Mamedov IZ, et al. (2015) MiXCR: software for comprehensive adaptive immunity profiling. Nat Methods 12: 380-381.

2. Nazarov VI, Pogorelyy MV, Komech EA, Zvyagin IV, Bolotin DA, et al. (2015) tcR: an R package for T cell receptor repertoire advanced data analysis. BMC Bioinformatics 16: 175.

3. McCoy AJ, Grosse-Kunstleve RW, Adams PD, Winn MD, Storoni LC, et al. (2007) Phaser crystallographic software. J Appl Crystallogr 40: 658-674.

4. Oomen CJ, Hoogerhout P, Kuipers B, Vidarsson G, van Alphen L, et al. (2005) Crystal structure of an Anti-meningococcal subtype P1.4 PorA antibody provides basis for peptide-vaccine design. J Mol Biol 351: 1070-1080.

5. Braden BC, Goldbaum FA, Chen BX, Kirschner AN, Wilson SR, et al. (2000) X-ray crystal structure of an anti-Buckminsterfullerene antibody fab fragment: biomolecular recognition of C(60). Proc Natl Acad Sci U S A 97: 12193-12197.

6. Emsley P, Lohkamp B, Scott WG, Cowtan K (2010) Features and development of Coot. Acta Crystallogr D Biol Crystallogr 66: 486-501.

7. Afonine PV, Grosse-Kunstleve RW, Echols N, Headd JJ, Moriarty NW, et al. (2012) Towards automated crystallographic structure refinement with phenix.refine. Acta Crystallogr D Biol Crystallogr 68: 352-367.

8. Shen Y, Maupetit J, Derreumaux P, Tuffery P (2014) Improved PEP-FOLD Approach for Peptide and Miniprotein Structure Prediction. J Chem Theory Comput 10: 4745-4758.

9. Bulheller BM, Hirst JD (2009) DichroCalc--circular and linear dichroism online. Bioinformatics 25: 539-540.

10. Tovchigrechko A, Vakser IA (2006) GRAMM-X public web server for protein-protein docking. Nucleic Acids Res 34: W310-314.

11. Sircar A, Gray JJ (2010) SnugDock: paratope structural optimization during antibody-antigen docking compensates for errors in antibody homology models. PLoS Comput Biol 6: e1000644.

12. Abraham MJ, Murtola T, Schulz R, Pall S, Smith JC, et al. (2015) GROMACS: High performance molecular simulations through multi-level parallelism from laptops to supercomputers. SoftwareX 1-2: 19-25.

13. Schmid N, Eichenberger AP, Choutko A, Riniker S, Winger M, et al. (2011) Definition and testing of the GROMOS force-field versions 54A7 and 54B7. Eur Biophys J 40: 843-856.
